# Rapamycin recruits SIRT2 for FKBP12 deacetylation during mTOR activity modulation in innate immunity

**DOI:** 10.1016/j.isci.2021.103177

**Published:** 2021-09-27

**Authors:** Lin Hu, Fuxian Chen, Chao Wu, Jun Wang, Si-si Chen, Xiang-rong Li, Jing Wang, Linpeng Wu, Jian-ping Ding, Jian-chuan Wang, Chao Huang, Hui Zheng, Yu Rao, Yu Sun, Zhijie Chang, Wei Deng, Cheng Luo, Y. Eugene Chin

**Affiliations:** 1 Institutes of Biological and Medical Sciences, Soochow University, 199 Ren’ai Road, Suzhou, Jiangsu 215123, China; 2 Drug Discovery and Design Center, State Key Laboratory of Drug Research, Shanghai Institute of Materia Medica, Chinese Academy of Sciences, Shanghai 201203, China; 3 Institute of Biochemistry and Cell Biology and Institute of Nutrition and Health Sciences, Chinese Academy of Sciences, 320 Yueyang Road, Shanghai 200031, China; 4 Program in Cellular and Molecular Medicine, Boston Children's Hospital, Boston, MA 02115, USA; 5 Laboratory of Membrane Biology, School of Medicine and School of Pharmaceutical Sciences, Tsinghua University, Beijing 100084, China; 6 Hematology center, cyrus Tang medical institute, Soochow University, 199 Ren’ai Road, Suzhou, Jiangsu 215123, China

**Keywords:** Biochemistry, Protein, Molecular biology

## Abstract

The mammalian target of rapamycin (mTOR) is a serine-threonine kinase involved in cellular innate immunity, metabolism, and senescence. FK506-binding protein 12 (FKBP12) inhibits mTOR kinase activity via direct association. The FKBP12-mTOR association can be strengthened by the immunosuppressant rapamycin, but the underlying mechanism remains elusive. We show here that the FKBP12-mTOR association is tightly regulated by an acetylation–deacetylation cycle. FKBP12 is acetylated on the lysine cluster (K45/K48/K53) by CREB-binding protein (CBP) in mammalian cells in response to nutrient treatment. Acetyl-FKBP12 associates with CBP acetylated Rheb. Rapamycin recruits SIRT2 with a high affinity for FKBP12 association and deacetylation. SIRT2-deacetylated FKBP12 then switches its association from Rheb to mTOR. Nutrient-activated mTOR phosphorylates IRF3S386 for the antiviral response. In contrast, rapamycin strengthening FKBP12-mTOR association blocks mTOR antiviral activity by recruiting SIRT2 to deacetylate FKBP12. Hence, on/off mTOR activity in response to environmental nutrients relies on FKBP12 acetylation and deacetylation status in mammalian cells.

## Highlights

• FKBP12-mTOR association is tightly regulated by an acetylation–deacetylation cycle
• SIRT2 is responsible for FKBP12 deacetylation
• Acetylation of Rheb is indispensable to mTOR activation
• mTOR phosphorylates IRF3 S386 for type-I interferon gene expression

## Graphical abstract

**Figure:**
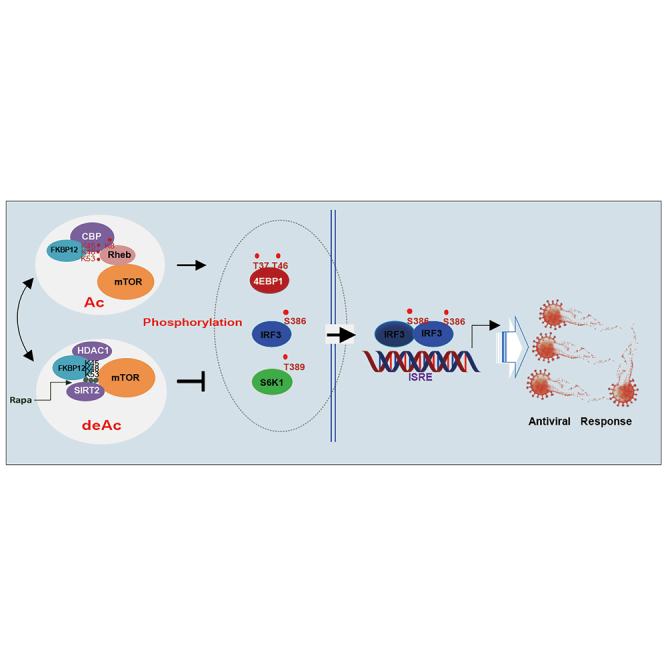

## Introduction

Mammalian target of rapamycin (mTOR) is a PI3K/PI4K type protein serine-threonine kinase in the cellular environment in mammalian cells that is activated in response to different stimuli. Nutrient or stress activated mTOR serves as the front line protein kinase for secondary line protein kinases or transcription factors activation, leading to diverse activities, including protein synthesis and gene regulation involved in senescence and innate immunity (Le Sage et al., 2016; Weichhart et al., 2015). In addition to the carboxyl-terminal catalytic domain, mTOR bears a number of regulatory domains within its NH_3_-terminal lobe. In mammalian cells, the mTOR kinase activity is regulated by a number of protein factors forming mTOR complex 1 (mTORC1) or mTOR complex 2 (mTORC2) according to their distinct core components and downstream signaling functions in a number of biological processes (Saxton and Sabatini, 2017). Among the mTORC1 kinase regulators, FK506-binding protein (FKBP) family member FKBP12 is one of the most prominent antagonists inhibiting mTOR catalytic activity (Hoeffer et al., 2008; Sabers et al., 1995; Chen et al., 1995). Although FKBP12 is a peptidyl-prolylcis-trans isomerase, inhibition of the enzymatic activity is irrelevant to immunosuppressive action of the drugs that bind to FKBP12 (Tropschug et al., 1990; Siekierka et al., 1989). FKBP12 directly binds to mTOR in the FKBP12-rapamycin binding (FRB) domain, which is adjacent to the catalytic domain of mTOR (Yang et al., 2013). Notably, rapamycin, an immunosuppressant drug, tightly associates with FKBP12 to inhibit mTOR-mediated cell signaling and impedes the growth and activity of immune cells.

mTOR can be activated by the small GTPase Rheb in response to amino acids or nutrient environments (Sancak et al., 2008; Long et al., 2005). Rheb can keep FKBP12 or other FKBP family members such as FKBP38 away and regulates mTOR catalytic activity in a guanosine 5′-triphosphate (GTP)-dependent manner (Bai et al., 2007). Rheb binds to mTOR directly, causing a global conformational change that allosterically reveals active-site residues and accelerates catalysis (Yang et al., 2017). The mTOR signalosome relies overall on serine-threonine phosphorylation for the downstream activation process. Deacetylase inhibitors such as trichostatin A (TSA) or nicotinamide (NAM) have been shown to modulate mTOR activity (Hong et al., 2014). Both Raptor and Rictor, the mTORC1 and mTORC2 components respectively, are under acetylation regulation in response to the cellular environmental changes (Glidden et al., 2012; Son et al., 2019). Since lysine deacetylase members physically interact with mTOR complex and lysine deacetylase inhibitors can modulate mTOR activity (Zhang et al., 2015; Ghosh et al., 2010; Back et al., 2011; Ma et al., 2015), mTOR signalosome activity is most likely under reversible acetylation modulation.

Apart from coordinating cell growth and metabolism with environmental inputs, mTOR has a master regulatory role in the innate immune system (Saemann et al., 2009). Innate immune responses, including antiviral responses, however, involve nuclear transcription regulation events. The presence of rapamycin in the cellular environment, specifically blocks mTOR activity for downstream substrates, such as the phosphorylation and activation of p70S6K and the release of eIF4E inhibition by PHAS-1/4E-BP1, as well as proinflammatory cytokines and type I IFNs involved in innate immune responses (Soliman, 2013; Digomann et al., 2019; Cao et al., 2008). Rapamycin was also demonstrated to impede the poly I:C-stimulated upregulation of INF-β expression in human oral keratinocytes (Zhao et al., 2010). There are some clues that cytoplasmic transcription factors, including MAPK and interferon regulatory factor (IRF) can be activated by mTOR during antiviral responses (Zhao et al., 2010; Fekete et al., 2014). Despite extensive studies, the mechanism by which mTOR regulates antiviral transcription events remains elusive.

Here, we found that FKBP12 and Rheb, the regulators of mTOR activity are precisely regulated by acetylation and deacetylation. CREB-binding protein CBP)-acetylated FKBP12 and Rheb undergo association. In contrast, only deacetyl-FKBP12 can then associate with mTOR. Both NAM (deacetylase sirtuin family inhibitor) and TSA (deacetylatase HDAC family inhibitor) dramatically promote the FKBP12-Rheb association but disrupt the FKBP12-mTOR association. Interestingly, rapamycin can recruit SIRT2 with high affinity, leading to FKBP12 deacetylation. In addition, we demonstrated that nutrient-activated mTOR induces IRF3 serine phosphorylation at S386. We provide evidence that mTOR can promote antiviral response by inducing IRF3 serine phosphorylation, whereas rapamycin blocks mTOR activity in the antiviral response via SIRT2 recruitment and SIRT2-mediated FKBP12 deacetylation.

## Results

### FKBP12 is acetylated in response to nutrient availability and is involved in regulating cellular viral infection activity

**Figure 1:**
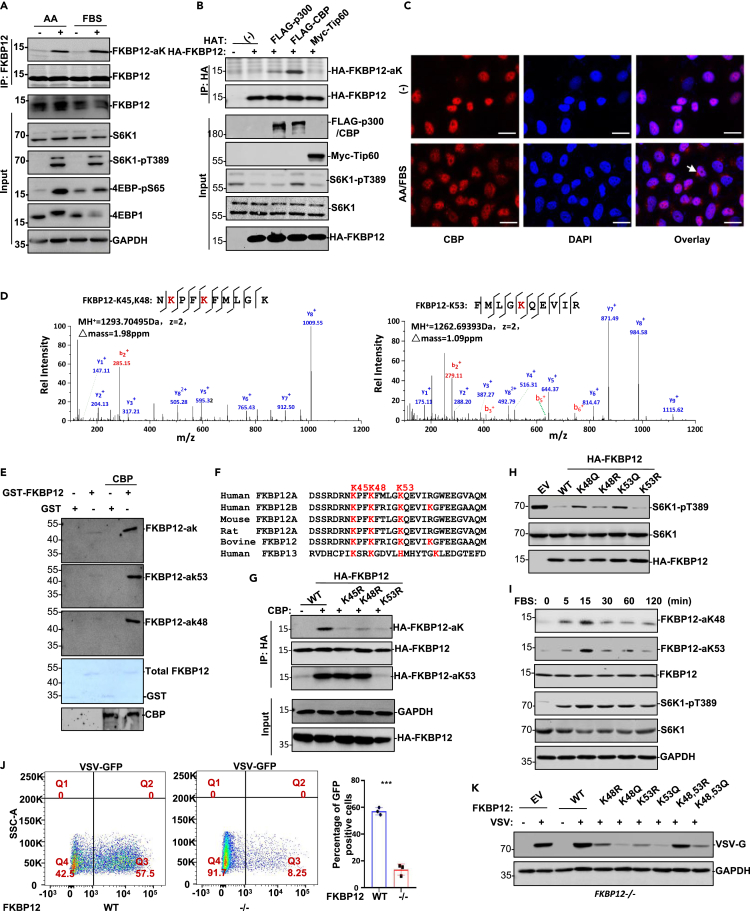
AA/FBS induces FKBP12 acetylation and involves in regulating cellular viral infection activity (A) Mouse fibroblasts starved for serum/AA for 24 h followed by AA or FBS treatment (30 min). FKBP12 immunoprecipitates prepared from WCL of these fibroblasts were analyzed in Western blot with pan anti-acetyl-K. (B) In HEK293T cells, HA-FKBP12 was cotransfected with p300, CBP or Tip60. FKBP12 immunoprecipitates were analyzed in Western blot with pan anti-acetyl-K. (C) Immunofluorescence staining of CBP with anti-CBP. DAPI was used for nuclear counter staining. Scale bar: 20 μm. (D) Acetyl-FKBP12 proteins were purified from HEK293T cells with Flag-FKBP12 and HA-CBP cotransfection. Mass spectrometry analysis of trypsinized acetyl-FKBP12 proteins uncovered K45, K48, and K53 as the acetyl-residues. (E) GST-FKBP12 recombinant protein was acetylated by CBP *in vitro*. (F) Alignment of the K-cluster of FKBP12 from different species. (G) FKBP12-K45R, -K48R, and -K53R variants were compared with wild type FKBP12 for acetylation induction by CBP in HEK293T cells with HA-FKBP12 transfected along with CBP. (H) HA-FKBP12 WT and its mutants were transiently transfected into 293T cells, WCLs were determined with the indicated antibodies. (I) As indicated, a time course to reveal FBS induced FKBP12 acetylation and S6K1 phosphorylation induction in FBS/AA starved mouse fibroblasts. Polyclonal antibodies against FKBP12-aK48 and FKBP12-aK53 were constructed for FKBP12 acetylation analysis. (**J**) WT or FKBP12−/− mouse fibroblasts infected with VSV, the infected cells were analyzed by flow cytometry. Left, representative image; right, statistical results of 3 independent experiments. Data are represented as mean ± SD; ∗∗∗, p < 0.001, two-tailed t test. (**K)** In FKBP12−/− mouse fibroblasts, indicated FKBP12 KR or KQ variants were overexpressed followed by VSV infection. VSV-G protein was analyzed in Western blot.

To investigate the mechanism by which FKBP12 impedes mTOR activity, we examined FKBP12 posttranslational modifications. mTOR was more active in FKBP12-depleted cells in both the presence and absence of rapamycin (Figure S1A), suggesting that mTOR activity can be regulated by endogenous FKBP12. FKBP12 acetylation was detected in the cytoplasmic fraction prepared from mouse fibroblasts that received amino acids or serum treatment (Figure 1A). Overexpression of CBP or its prolog p300 in HEK293T cells induced FKBP12 acetylation (Figure 1B). CBP is mainly a nuclear factor that can be exported from nuclei in response to various stimulations (Tang et al., 2007; Cohen et al., 2004). Nutrient (amino acids and serum) supplementation induced CBP nuclear exportation in HeLa cells (Figure 1C). Given that CBP was the most prominent acetyltransferase in FKBP12 acetylation induction in cytoplasm, we purified CBP-acetylated FKBP12 from HEK293T transfectants for mass spectrometry analysis, and the results showed that three lysine residues (K45, K48, and K53) of the FKBP12 central lysine cluster were acetylated by CBP (Figures 1D and S1B). Specific polyclonal antibodies recognizing acetyl-K48 and acetyl-K53 of FKBP12 were then prepared (Figure S1C). Upon *in vitro* acetylation with recombinant CBP protein, GST-FKBP12 recombinant protein was recognized by the pan-Ack, acetyl-K48 and acetyl-K53 antibodies (Figure 1E). SGC-CBP30, a selective CBP/p300 bromodomain inhibitor, suppressed FKBP12 acetylation (Figure S1D). These data indicate that FKBP12 is acetylated by CBP both *in vitro* and *in vivo*. These lysine residues are highly conserved in the FKBP12 family from different species (Figure 1F). Site-directed mutagenesis of the FKBP12 lysine cluster confirmed that all three conserved lysine residues (K45, K48, and K53) were acetylated by CBP (Figure 1G).

FKBP12 associates with mTOR to restrict its catalytic activity. Therefore, we next tested the effect of KR or KQ mutated FKBP12 on mTOR activity regulation. FKBP12 with the KR mutation, which is a mimic of nonacetylated lysine (KR mutant) largely restricted mTOR activity in S6K1 phosphorylation induction, whereas FKBP12 with the KQ mutation, which is a mimic of acetyl lysine (KQ mutant) became weaker in mTOR activity inhibition than wild-type or KR mutants of FKBP12 (Figure 1H). The time course of serum treatment revealed that FKBP12 acetylation in fibroblasts was induced in 5 min and reached a maximum in 15 min followed by a decay of acetylation intensity (Figure 1J). Correspondingly, phosphorylation induction of the mTOR substrate S6K1 by serum showed a similar pattern (Figure 1I). AKT and PKCα activity, however, did not change in response to CBP overexpression in HEK293T cells (Figure S1E), suggesting that mTORC1 complex activity is more sensitive than mTORC2 complex activity in CBP-mediated mTOR activity regulation. To investigate how FKPB12 acetylation affects mTOR function, FKBP12−/− fibroblasts were infected with VSV, and flow cytometry analysis revealed that the efficiency of infection was severely decreased in FKBP12−/− cells (Figure 1J). In addition, wild-type FKBP12 or the lysine cluster variants were reintroduced into FKBP12−/− fibroblasts to compare their response to the subsequent VSV infection, the results showed that KQ mutation of the lysine cluster largely abolished viral protein production (Figure 1K).

### Rapamycin recruits SIRT2 with high affinity to deacetylate FKBP12, whereas HDAC members deacetylate FKBP12 via a distinct mechanism

**Figure 2:**
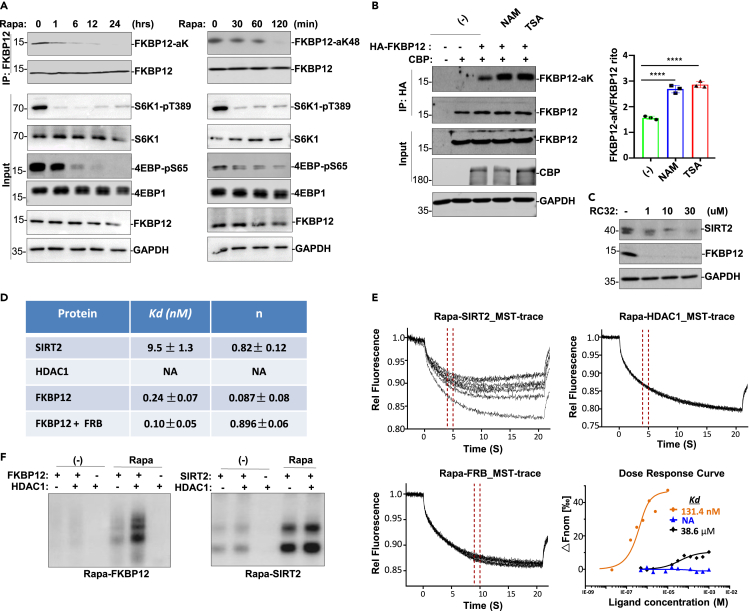
SIRT2 deacetylates FKBP12 (A) FKBP12 deacetylation induction by rapamycin in mouse fibroblasts for indicated times and blotted with indicated antibodies. (B) In HEK293T cells, HA-FKBP12 and FLAG-CBP were cotransfected followed by NAM, or TSA treatment as indicated. Anti-HA IP was analyzed for FKBP12 acetylation with pan acetyl-K antibody in Western blot. FKBP12-acK band intensities were measured by ImageJ software and normalized to FKBP12 (right panel). Data are represented as mean ± SD; ∗∗∗∗, p < 0.0001, one-way ANOVA. (C) HeLa cells were treated with indicated doses of RC32 for 12 h. WCLs were analyzed for SIRT2 or FKBP12 degradation with indicated antibodies. (D) Rapamycin binding affinity Kds were obtained from ITC analysis of indicated proteins purified from bacteria. (E) Purified SIRT2, HDAC1, and FRB proteins were incubated with rapamycin for MST analysis. (F) Naïve PAGE detection of rapamycin ternary complex formation with FKBP12 (left) or with SIRT2 (right). GST-FKBP12, GST-SIRT2 and GST-HDAC1 were purified from bacteria and incubated with rapamycin prior to loading onto the naive PAGE and blotted with anti-GST.

We next determined which deacetylases are responsible for FKBP12 regulation. Rapamycin treatment induced FKBP12 deacetylation in a time dependent manner in fibroblasts (Figure 2A). However, rapamycin did not affect CBP expression and activity in HEK293T cells (Figures S2A and S2B), suggesting that the deacetylation effect of rapamycin on FKBP12 was not due to an inhibitory effect on CBP but rather to changes in deacetylase enzymatic activity. Indeed, both nonspecific deacetylase inhibitors NAM and TSA treatments enhanced FKBP12 acetylation induction by CBP (Figure 2B), indicating that NAD^+^-dependent sirtuin-type and HDAC-type deacetylases are involved in FKBP12 deacetylation regulation. By screening all seven sirtuin family members, each was individually transfected along with FKBP12 and CBP followed by rapamycin treatment or not, in HEK293T cells, we found that CBP-acetylated FKBP12 was primarily deacetylated by SIRT2 or its closely related family member SIRT1 (Figures S2C and S2D). The catalytically inactive form of SIRT2(H187Y) largely abolished FKBP12 deacetylation induction (Figure S2E), and FKBP12 was constitutively acetylated at K48 (Figure S2F) in *SIRT2*^*−/−*^ mouse fibroblasts, all supporting a critical role for SIRT2 in regulating FKBP12 deacetylation. We also uncovered that SIRT2 preferentially deacetylates FKBP12 at K48 by differential tandem mass tag analysis using a peptide containing the acetyl-K48 sequence of FKBP12 (Figure S2G).

To confirm a direct interaction between rapamycin and SIRT2, we tested SIRT2 stability with the compound RC32, which conjugates rapamycin and pomalidomide for FKBP12 degradation (Sun et al., 2019). RC32 caused SIRT2 degradation while depleting FKBP12 in HeLa cells (Figure 2C), suggesting a direct association between rapamycin and SIRT2. We also tested whether rapamycin can inhibit mTORC1 signaling after cells were treated with RC32, and the results showed that indeed rapamycin cannot inhibit mTORC1 when cells are treated with RC32 (Figure S2H). We next performed a group of *in vitro* experiments to verify the binding of rapamycin with SIRT2. Recombinant SIRT2, FKBP12, FRB, and HDAC1 proteins purified from bacteria were incubated with rapamycin individually or in combination at different concentrations, and the dissociation constant was determined by isothermal titration calorimetry (ITC) assay and microscale thermophoresis (MST) assay (Figures 2D and 2E). In ITC, the heat transfer during binding is accurately measured to determine the binding constants and other thermodynamic parameters. ITC results showed that the rapamycin and FKBP12 binding *Kd* was 0.24 nM, whereas in the presence of FRB, the FKBP12 and rapamycin binding *Kd* was 0.10 nM, agreeing with previous reports that FKBP12 and rapamycin binding *Kd* was 0.2 nM and FKBP12 and rapamycin binding *Kd* in the presence of FRB was 0.1 nM (Banaszynski et al., 2005; Bayle et al., 2006). The rapamycin and SIRT2 binding *Kd* was 9.5 nM (Figure 2D). No *Kd* was fitted from the rapamycin and HDAC1 association, apparently due to their poor, or no, interaction. MST measures the thermophoresis of molecules in a laser-induced temperature gradient. In the MST assay, the rapamycin and SIRT2 binding *Kd* was 131 nM and the rapamycin and FRB binding *Kd* was 38.6 nM (Figure 2E). No direct binding was detected between rapamycin and HDAC1, even though rapamycin treatment induced both HDAC1-FKBP12 (Figure S2I, left panel) and SIRT2-FKBP12 associations (Figure S2I, right panel). Although the *Kd* values measured in these two assays were different, both measurements validated that SIRT2 is a specific substrate of rapamycin. We also incubated rapamycin with purified FKBP12 or HDAC1 alone or together and submitted it to naive polyacrylamide gel electrophoresis (PAGE) for analysis. HDAC1 proteins dramatically enhanced rapamycin and FKBP12 association. Under the same conditions used, HDAC1 proteins failed to affect the SIRT2 and rapamycin association (Figure 2F).

### FKBP12 in acetylated and deacetylated states modulates mTOR activity

**Figure 3:**
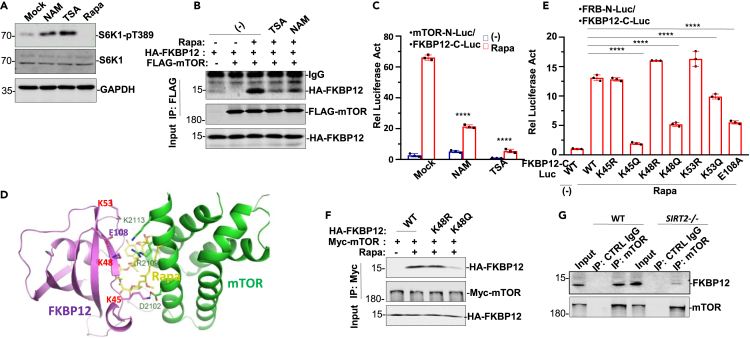
Deacetylated-FKBP12 associates with mTOR (A) Fibroblasts were treated with NAM, TSA or rapamycin for 1 h, WCLs were prepared for Western blot analysis S6K1 activation. (B) In HEKT293T cells, HA-FKBP12 was transfected with or without Flag-mTOR as indicated followed by no treatment or rapamycin, rapamycin and TSA or rapamycin and NAM cotreatment. Anti-Flag IP was analyzed for HA-FKBP12 and Flag-mTOR interaction in Western blot with anti-HA. (C) Dual-elements reporter system i.e. mTOR (FRB)-N-Luciferase reporter and FKBP12-C-luciferase reporter were cotransfected in HEK293T cells followed by rapamycin treatment for 6 h. Pretreatment with TSA or NAM for 2 h significantly blocked mTOR and FKBP12 interaction induced by rapamycin. Data are represented as mean ± SD; ∗∗∗∗, p < 0.0001, one-way ANOVA. (D) The interface between FKBP12 (purple) and mTOR FRB domain (green) in their interaction. Rapamycin was in yellowish. interaction interface with mTOR. While FKBP12 K45 interacts directly with mTOR D2102 to form salt bond, FKBP12 K48 forms salt bond with FKBP12 E108, ∗∗∗∗p < 0.0001, one-way ANOVA. (E) In HEK293T cells, mTOR-N-Luc was transfected along with FKBP12-C-Luc (WT or various mutants as indicated) were cotransfected followed by rapamycin treatment. The luciferase activity was analyzed. Data are represented as mean ± SD; ∗∗∗∗, p < 0.0001, one-way ANOVA. (F) In HEK293T cells, HA-FKBP12 WT and variants (K48R and K48Q) were transfected along with mTOR. Anti-Myc IP was analyzed for HA-FKBP12 and Myc-mTOR interaction in Western blot using anti-Myc. (G) In fibroblasts obtained from WT or SIRT2−In mouse, mTOR IP was analyzed for FKBP12 interaction in Western blot.

Both NAM and TSA treatments elevated S6K1 phosphorylation in fibroblasts (Figure 3A). AA/serum starvation enhanced the FKBP12 and mTOR association in response to rapamycin treatment (Figure S3A). However, both NAM and TSA treatment readily abolished the association of FKBP12 and mTOR in response to rapamycin (Figure 3B). Thus, sirtuin and HDAC family members are involved in modulating the FKBP12-mTOR association and FKBP12 activity even though HDAC family members do not directly interact with rapamycin. Although rapamycin promotes the already effective association between FKBP12 and mTOR, the disruptive effect of NAM or TSA on the FKBP12-mTOR association was confirmed using an optimized mTOR FRB-N-terminal fragment (N-Luc) and C-terminal fragment (C-Luc)-FKBP12 dual element luciferase assay, which was extraordinarily sensitive in characterizing FKBP12 and mTOR association/dissociation in mammalian cells (Figure 3C) (Paulmurugan and Gambhir, 2007). Compared with specific HDAC inhibitors (HDAC1-3 inhibitor CI-994, HDAC1/HDAC2 inhibitor FK-228, HDAC3 inhibitor RGF966, HDAC4/HDAC5 inhibitor LMK225, and HDAC6 inhibitor nexturastat A) (Monneret, 2005), the nonspecific HDAC inhibitor TSA was still the most effective at inhibiting rapamycin-induced FKBP12-mTOR complex formation (Figure S3B), indicating that multiple HDAC family members are capable of deacetylating FKBP12. Structural analysis of the protein-protein interaction between FKBP12 and mTOR revealed that the K45, K48, and K53 residues reside in the interface of the direct association with mTOR (Figure 3D). Among the three residues, K45 of FKBP12 is capable of hydrogen bounding directly with D2102 of mTOR, whereas K48 and K53 can be important in allosteric regulation of FKBP12 conformation required for mTOR interaction in the presence of rapamycin (Figure 3D) (Yang et al., 2013). FKBP12 with KQ mutation of the three cluster lysine residues as well as K48 interacting residue E108 mutant (E108A) reduced FKBP12’s association with mTOR (Figure 3E), in agreement with the 3-dimensional-structural analyses of FKBP12-mTOR complex that the lysine cluster of FKBP12 forms the interface for FKBP12 to interact with mTOR. Co-immunoprecipitation (co-IP) of FKBP12 and mTOR revealed that FKBP12 with KQ but not KR mutation of K48 or K53 residue markedly attenuated the FKBP12-mTOR association (Figures 3F and S3C). In *SIRT2−/−* fibroblasts, the FKBP12 and mTOR association was disrupted (Figure 3G), apparently due to constitutive acetylation of FKBP12 in fibroblasts lacking SIRT2 expression (Figure S2E).

### Rheb is also acetylated during nutrient treatment and contributes to mTOR activation

**Figure 4:**
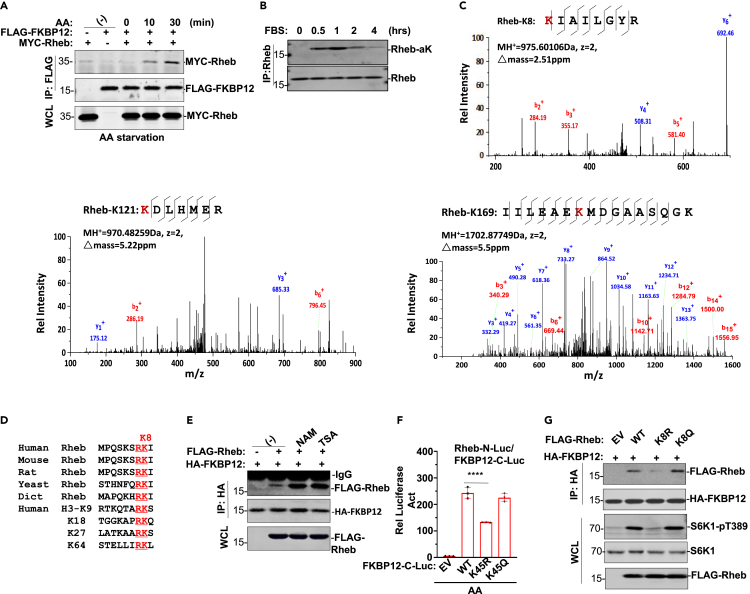
Acetylated-FKBP12 associates with acety-Rheb contributing to mTORC1 kinase activation (A) HEK293T cells transfected with FLAG-FKBP12 and Myc-Rheb followed by AA treatment for indicated times. Anti-Flag IP was analyzed for FKBP12 and Rheb interaction in Western blot. (B) In fibroblasts, 20% FBS treatment for indicated time. WCLs prepared for Rheb IP followed by pan acetyl-K blotting. (C) Mass spectra of acetyl-K8, acetyl-K169 and acetyl-K120 peptides of Rheb recovered from trypsin digests. (D) Alignment of double-positive “RK” motif of Rheb of different species with “RK” motifs of histone H3. (E) HA-FKBP12 and Myc-Rheb were cotransfected in HEK293T cells. NAM or TSA treatment for 2 h was tested on FKBP12 and Rheb interaction. (F) Two reporter system (Rheb-N-Luc and FKBP12-C-Luc of WT, K48R or K48Q) were cotransfected and treated with rapamycin treatment for 6 h followed by the luciferase reporter activity analysis. Data are represented as mean ± SD; ∗∗∗∗, p < 0.0001, one-way ANOVA. (G) Flag-Rheb WT or variant (K8R, K8Q) or EV was cotransfected with HA-FKBP12 in HEK293T cells. Anti-HA IP was analyzed for FKBP12 and Flag-Rheb interaction in Western blot with anti-Flag. WCLs were analyzed for S6K1 phosphorylation with anti-S6K1 pT389.

Although Rheb is an important activator of mTORC1, exactly how it stimulates mTORC1 kinase activity remains elusive. Treatment of HEK293T cells with amino acids or serum for up to 30 min induced FKBP12 and Rheb association (Figures 4A and S4A), supporting the previous finding that FKBP12 family members interact with Rheb, the KRAS-like GTPase for mTOR kinase activation during mTORC1 signaling (Bai et al., 2007). Rheb was also acetylated in fibroblasts in response to nutrient supplementation (Figure 4B) and in HEK293T cells by ectopic CBP overexpression (Figure S4B). K8, K121, and K169 residues were identified as acetylation sites on Rheb purified from HEK293T transfectants (Figures 4C and S4C). Rheb was also acetylated by CBP *in vitro* (Figure S4D). Moreover, the Rheb K8 residue with the “RKX” sequence is species conserved (Figure 4D). “RKX” motifs, i.e., acetyl-“RKS” or acetyl-“RKΨ” motifs (H3K18: RKQ or H3K79: RKL) in histone H3 N-tail, provide various enzymes or transcription factors for association during epigenetic gene expression regulation (Tang et al., 2007; Fischle et al., 2005).

Thus, the central lysine cluster of FKBP12 tightly modulates the FKBP12-mTOR association during rapamycin treatment. NAM or TSA treatment greatly enhanced FKBP12 and Rheb complex formation (Figure 4E), suggesting that acetylation was involved in the interaction between these two proteins. The K45 residue of FKBP12 was involved in the interaction between FKBP12 and Rheb, and FKBP12 with the K45R mutation but not the K45Q mutation reduced its association with Rheb (Figure 4F). We therefore conclude that FKBP12 switches its association between mTOR and Rheb largely depending on its deacetylation or acetylation status. The Rheb-K8R variant reduced Rheb’s interaction with FKBP12, whereas Rheb-K8Q had the opposite effect (Figures 4G and S4E). Equivalently, the Rheb-K8R variant largely abolished mTOR activity in S6K1 phosphorylation, while Rheb-K8Q did not (Figure 4G). In comparison, neither the K121R nor the K169R mutation of Rheb affected mTOR activity or the FKBP12-mTOR association (Figures S4F–S4G), suggesting that Rheb acetylation at K8 residue plays a central role in mTOR activation. In addition, The interaction of Rheb with acetylated FKBP12 seem to be dependent on its nucleotide binding states because GTP loaded Rheb exhibited much higher binding affinity toward acetylated FKBP12 than did GDP loaded or untreated Rheb (Figure S4H).

### mTOR phosphorylates IRF3 S386 for INFs induction

**Figure 5:**
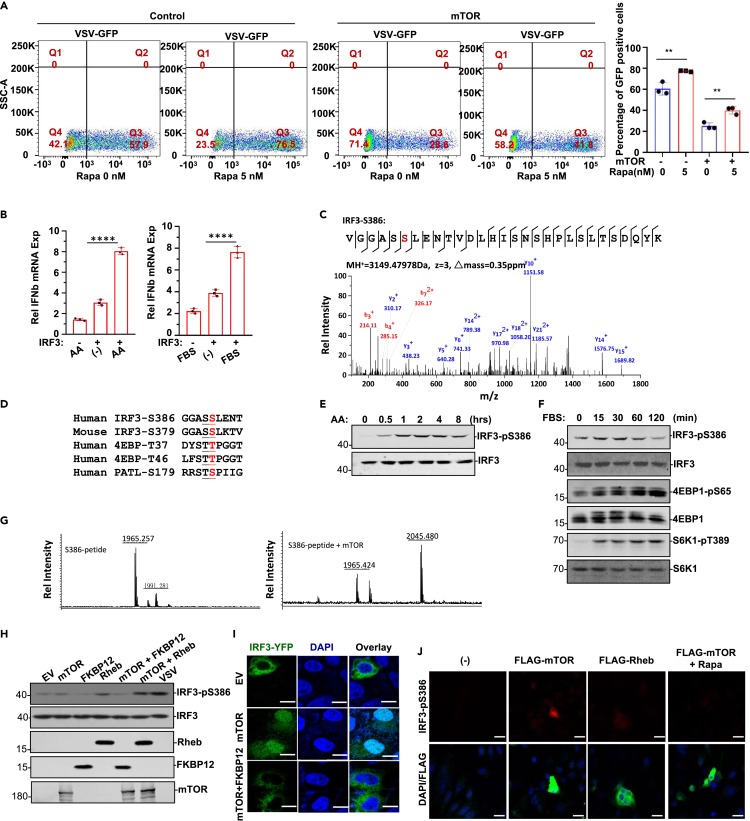
IRF3 S386 phosphorylation by mTOR is for antiviral genes expression (A) The mTOR stable HeLa cell line and control cell line were treated with or without 5 nM Rapamycin for 24 h, then the cells infected with VSV, the infected cells were analyzed by flow cytometry. Left, representative image; right, statistical results of 3 independent experiments. Data are represented as mean ± SD; ∗∗, p < 0.01, two-tailed t test. (B) HEK293T cells were transfected with or without IRF3 as indicated followed by AA treatment or FBS treatment. RT-PCR was performed to analyze IFNα mRNA expression level. Data are represented as mean ± SD; ∗∗∗∗, p < 0.0001, one-way ANOVA. (C) IRF3 protein S386 phosphorylation identified with mass spectrometry. IRF3 was purified from HEK293T cells transfected with IRF3 along with mTOR. (D) IRF3 S385S386 motif was aligned with T36T37 and T45T46 motifs of 4EBP. (E) WCLs were prepared from HeLa cells receiving AA treatment for indicated times. IRF3 phosphorylation was then analyzed in Western blot with anti-IRF3-pS386. (F) WCLs were prepared from mouse fibroblasts receiving FBS treatment for indicated times. Anti-IRF3-pS386, anti-4EBP1-pS65, and anti-S6K1-pT389 were applied in Western blot. (G) Modi-TOF analysis of S386 peptide (ARVGGASSLENTVDLHI) phosphorylation by mTOR *in vitro*. (H) Western blot analysis revealed that while Rheb elevated mTOR in IRF3 phosphorylation induction, FKBP12 attenuated its activity in HEK293T cells. VSV infection induced IRF3 phosphorylation on S386 in fibroblasts. (I) GFP-IRF3 was cotransfected with EV, mTOR, or mTOR + FKBP12 in Hela cells. GFP and DAPI staining were visualized with confocal fluorescent microscope. mTOR overexpression caused IRF3 nuclear translocation whereas FKBP12 cotransfecteion with mTOR blocked IRF3 nuclear translocation. Scale bar: 10 μm. (J) In Hela cells, EV, Flag-mTOR, or Flag-Rheb was transiently transfected followed by rapamycin treatment, Upper panel was immune-stained with anti-IRF3-pS386. The lower panel was an overlay of DAPI staining and anti-Flag immune-staining. Scale bar: 20 μm.

mTOR directly regulates IRF3 activity in the antiviral response and IFN production (Bodur et al., 2018; Fekete et al., 2014; Ohman et al., 2015). Rapamycin can facilitate viral replication by regulating mTOR activity (Zhou et al., 2014; Alain et al., 2010). Upon 5 nM rapamycin treatment, VSV infection was elevated in HeLa cells stably overexpressing empty vector and mTOR (Figure 5A), suggesting that rapamycin enhances VSV replication by mTOR. Amino acids or FBS treatment greatly induced IFNα or IFNβ gene expression in HEK293T cells transfected with or without IRF3 (Figures 5B and S5A). Our mass analysis revealed that IRF3 was phosphorylated by mTOR on S386 within the MH2 domain (Figures 5C and S5B). Intriguingly, mTOR preferentially phosphorylates the second serine/threonine residue of those serine/threonine duplicates, such as S386 of S385/S386 of the IRF3-MH2 domain and T37 of T36/T37 and T47 of T46/T47 in the 4EBP factor (Figure 5D). It is worth noting that a supershifting band in the Western blot of IRF3 was observed upon mTOR overexpression, indicating that mTOR might phosphorylate not only S386 but also other sites. Indeed, IRF3 has been reported to be phosphorylated by mTOR at other serine sites (Figure S5C) (Saemann et al., 2009). IRF3-S386 phosphorylation was induced by serum or amino acids treatment in a time-dependent manner (Figures 5E and 5F). Obviously, mTOR not only induced phosphorylation of S6K1 at T389 and 4EBP1 at S65, but also induced IRF3 sphosphorylation at S386 in response to serum treatment (Figure 5F). We confirmed that mTOR phosphorylates IRF3 S386 by *in vitro* phosphorylation experiment (Figure S5D). In addition, rapamycin treatment inhibited IRF3-S386 phosphorylation in fibroblasts (Figure S5E). Utilizing purified Flag-mTOR protein to incubate with synthesized IRF3-S386 peptide under *in vitro* kinase assay conditions, IRF3 S386 phosphorylation by mTOR was confirmed by mass spectrometric analysis (Figure 5G). In VSV infected cells, IRF3-S386 phosphorylation was induced, reflecting the host cell response to viral infection (Figure 5H). IRF3-S386 phosphorylation by mTOR was enhanced by cotransfection of IRF3 with Rheb but reduced by cotransfection of IRF3 with FKBP12 (Figure 5H). IRF3 underwent nuclear translocation when mTOR was overexpressed alone but not when mTOR and FKBP12 were expressed together (Figure 5I). Moreover, IRF3 S386 phosphorylation induction by mTOR overexpression was elevated by Rheb but inhibited by FKBP12 cotransfection (Figure S5F). Immunofluorescence experiments showed that Rapamycin treatment blocked pS386-IRF3 nuclear induction and translocation (Figure 5J). These results suggest that mTOR is a mediator of gene regulation in both host responses and virus countermeasures.

### Acetyl-mTORC1 complex is required for IRF3 S386 phosphorylation to promote antiviral activity

**Figure 6:**
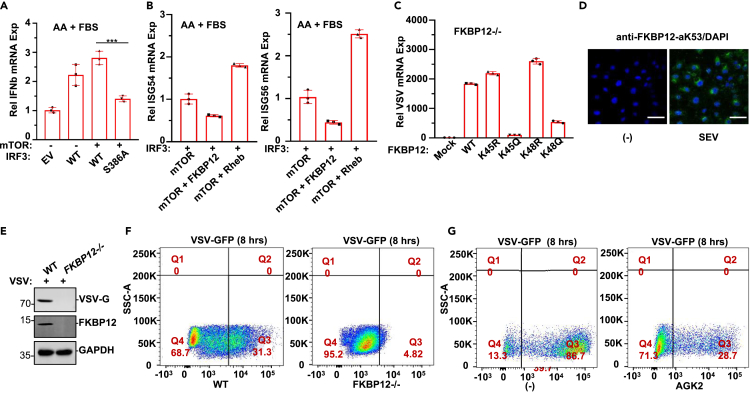
mTOR-IRF3 pathway triggers antiviral activity (A) HEK293T cells were transfected with or without IRF3 (WT and S386A) as indicated followed by amino acids (AA) or fetal bovine serum (FBS) treatment. RT-PCR was performed to analyze IFNβ mRNA expression level. Data are represented as mean ± SD; ∗∗∗, p < 0.001, one-way ANOVA. (B) In HEK293T cells, IRF3 was cotransfected with mTOR alone or combined as indicated. ISG54 and ISG56 mRNA expression were analyzed with RT-PCR. Data are represented as mean ± SD. (C) In FKBP12−/− mouse fibroblasts, FKBP12-K45R, -K45Q, -K48R, and -K48Q variants were overexpressed followed by VSV infection. RT-PCR was performed to analyze VSV expression. Data are represented as mean ± SD. (D) In mouse fibroblasts with SEV or no virus infection, anti-FKBP12-aK53 immunostaining and DAPI staining were visualized with confocal fluorescent microscope. Scale bar: 20 μm. (E) Western blot analysis revealed that VSV protein product was only detected in WT but not in FKBP12−/− cells. (F) WT or FKBP12−WT mouse fibroblasts infected with VSV, the infected cells were analyzed by flow cytometry. (G) Mouse fibroblasts were infected with VSV-GFP followed by AGK2 (20 μM) treatment and the cells were analyzed with flow cytometry.

We next sought to understand the mechanism by which mTORC1 modulates IRF3 for antiviral activity. We first tested the role of IRF3 S386 phosphorylation in gene expression. RT-PCR showed that IRF3-S386 phosphorylation by mTOR was critical in type I IFN gene IFN-β expression induction (Figure 6A). mTOR activity in antiviral gene (ISG54 and ISG56) expression induction was elevated by cotransfecting mTOR with Rheb but restricted by FKBP12 (Figure 6B). IFN-β mRNA levels and ISRE-element luciferase activity were then analyzed by overexpressing mTOR, FKBP12, and Rheb in HEK193T cells. The results of both IFN-β mRNA induction and ISRE-element luciferase reporter activity revealed that IRF3 overexpression, along with mTOR enhanced IRF3 transcriptional activity (Figures S6A and S6B).

The antiviral response of the FKBP12-mTOR route was also analyzed in human 2fTGH fibroblasts. The viral gene products UL46 and ICP27 of herpes simplex virus (HSV) and VSV-G of VSV were all elevated when FKBP12 was cotransfected along with mTOR and IRF3 (Figure S6C). FKBP12 with the KQ mutation but not the KR mutation of the lysine cluster largely abolished viral mRNA expression (Figure 6C). As expected, viral infection induced FKBP12 acetylation on the K53 residue in fibroblasts (Figure 6D). In *FKBP12−/−* cells infected with VSV or HSV, the viral gene product VSV-G protein or UL42 protein was undetectable (Figures 6E and S6D). Consequently, the virus was highly duplicated in wild-type cells but not in *FKBP12−/−* cells (Figure 6F). HSV is a large nuclear-replicating DNA virus, which activates PI3K (Prejean et al., 2001). Most notably, HSV degraded IFN-β, but not cGAS or STING, in line with prior reports, and this occurred irrespective of US3 activity (Kalamvoki and Roizman, 2014). The SIRT2 inhibitor AGK2 dramatically blocked VSV infection in fibroblasts (Figures 6G and S6E). Acetylation of FKBP12 blocked viral proliferation, whereas deacetylated FKBP12 supported viral proliferation by blocking mTOR activity during innate immunity induction. Taken together, the FKBP12-mTOR forms the deacetyl-mTORC1 complex activity in the presence of SIRT/HDAC deacetylases promote viral replication in cells, whereas Rheb-FKBP12 forms acetyl-mTORC1 complex activity in the presence of CBP prevents viral replication in cells, consistent with their actions within a common pathway in response to viral stress.

**Figure 7:**
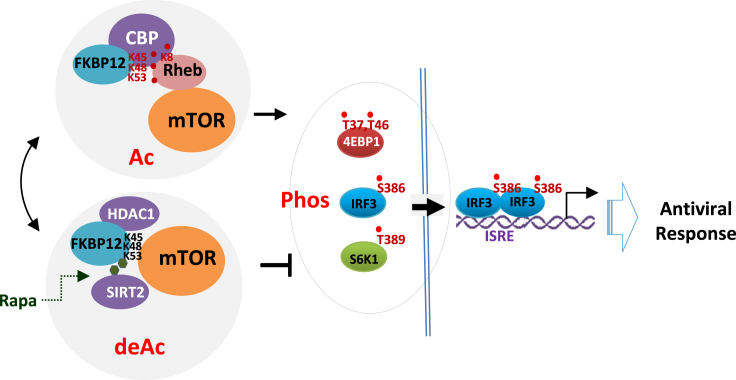
Illustration of Rapamycin-SIRT2 dependent FKBP12-mTOR inactivation in innate immunity FKBP12 can be acetylated in cells in the presence of nutrients. Rapamycin recruits SIRT2 but not HDAC1 to deacetylate FKBP12, which can then associate with mTOR. Acetyl-FKBP12 however associates with acetyl-Rheb to form a moderate mTOR activator. mTOR activated IRF3 undergoes nuclear translocation and gene activation leading to anti-viral response, which can be restricted by rapamycin treatment.

Given that HAT and deacetylases are essential components of mTOR signalosome, mTOR activity is under reversible acetylation modulation in cells (Figure 7).

## Discussion

The catalytic activity of mTOR in serine/threonine phosphorylation is under tight enzymatic regulation by forming an mTORC1 complex with small protein enzymes, including FKBP12 and Rheb. To keep mTOR in an active form, FKBP12 and Rheb need to recruit the acetyltransferase CBP for acetylation induction. In contrast, FKBP12 recruits deacetylase to maintain its deacetylation status to inhibit mTORC1 complex activity. Acetyl-Rheb attenuates the negative effect of FKBP12 by associating with acetyl-FKBP12. Lysine acetyltransferase (KAT) and lysine deacetylase (KDAC) complexes are involved in mTORC1 and mTORC2 complex activity regulation (Ma et al., 2015; Wan et al., 2017). Raptor is acetylated by EP300 on the K1097 residue. Decreased acetyl-raptor inhibits mTORC1 and EP300 activity in fasted mouse tissues during mTORC1 regulation by Leu metabolism (Lundby et al., 2012; Son et al., 2019). TSC2 in its acetylated state is linked to ubiquitin ligation and degradation, leading to mTORC1 activation and cell proliferation. TSC2 deacetylation is suspected to induce autophagy (Garcia-Martinez and Alessi, 2008). Intriguingly, we showed that SIRT2 and HDAC1 are recruited by rapamycin and FKBP12, respectively, leading to FKBP12 deacetylation. The FKBP12 acetylation event is a precise regulation process in control mTORC1 signaling. This process is in a dynamic equilibrium, in which acetylation and deacetylation reactions occur continuously, generating a steady-state level of FKBP12. Rapamycin preferentially associates with the beta sheet loop of FKBP12 (Liang et al., 1999). SIRT2 is helical overall in structure, which is highly homologous to that of the FRB domain involved in association with rapamycin-FKBP12 (Finnin et al., 2001). However, the underlying mechanisms still need to be further explored. In contrast, in the absence of rapamycin, KAT, such as CBP/p300 acetylates FKBP12 and Rheb to form a catalytically active acetyl-mTOR1 complex.

The interface of FKBP12 for interaction with mTOR bears this conserved lysine cluster, which is now acetylation–deacetylation dependent for its conformational change. Lysine cluster acetylation does not affect its rapamycin association. Ras homolog enriched in brain (Rheb) GTPase directly interacts with mTOR in GTP-bound form and strongly stimulates its kinase activity. Although Rheb may not be directly involved in the AAs-sensing mechanism of mTORC1, it is absolutely required for mTORC1 activation by AAs (Jewell et al., 2013; Sancak et al., 2008). In Rheb knockdown cells, AAs are ineffective in stimulating mTORC1 (Hall et al., 2007). Conversely, mTORC1 is active in cells overexpressing constitutively active or wild-type Rheb, which has much higher basal GTP binding than other GTPases, such as Ras, even under AA starvation, suggesting that Rheb acts either downstream or parallel to AA (Saucedo et al., 2003). We showed here that Rheb bound with more affinity to acetylated FKBP12 than to nonacetylated FKBP12 after the addition of AA, whereas dissociated from nonacetylated FKBP12 without AA or rapamycin treatment. The mechanism for the differential binding affinity of Rheb to acetylated or nonacetylated FKBP12 is currently unknown; however, it may involve different statuses of Rheb that bind GTP or GDP.

Viruses can utilize host cell intrinsic machinery for replication and trigger cell intrinsic stress responses that restrict viral reproduction (Goubau et al., 2013; Meade et al., 2018). The acetyl-mTORC1 complex and deacetyl-mTORC1 complex represent two different statuses of the mTORC1 complex in cells in response to environmental stresses. As such, viruses counter cytosolic sensing through a unique strategy of directly targeting and dysregulating the mTORC1 complex. Therefore, CBP is involved in mTORC1 complex formation, leading to mTOR activation. In contrast, HDAC1 and SIRT2 are involved in deacetyl-mTORC1 complex formation, leading to mTOR activity shutdown. Although the formation of a tripartite S6K1-STING-TBK1 complex was previously reported for the activation of IRF3 (Wang et al., 2016), short-term rapamycin treatment produced profound inhibition of mTOR-dependent gene expression activities, correlating with a pro-viral response (Meade et al., 2018). We found that mTOR induced an antiviral response directly via IRF3-S386 phosphorylation. Thus, rapamycin-SIRT2 promoted FKBP12-mTOR association and disrupted IRF3 activation by mTOR for the antiviral response.

### Limitations of the study

This study demonstrated that mTORC1 activity relies on FKBP12 acetylation and deacetylation status in response to growth signals. In addition, activated mTOR phosphorylates IRF3 S386 for the antiviral response. The questions such as (1) which specific growth signals stimulate the acetylation of FKBP12, (2) how acetyl-FKBP12 associates with acetylated Rheb to regulate mTORC1 activity precisely, and (3) whether the phosphorylation of IRF3 S386 site by mTOC1 may be interfered by TBK1 in physiological condition remain to be investigated in the future.

## STAR★Methods

### Key resources table

**Table:** 

REAGENT or RESOURCE	SOURCE	IDENTIFIER
**Antibodies**		

Anti-lys-48 FKBP12	This paper	N/A
Anti-lys-53 FKBP12	This paper	N/A
anti-FKBP12	Santa Cruz	CAT:sc-133067; RRID:AB_2102847
anti-Myc	Santa Cruz	CAT: sc-40; RRID:AB_2857941
anti-HA	Santa Cruz	CAT: sc-7392
anti-phosphoS6K Thr389	Cell Signaling Technology	CAT:#9234S; RRID:AB_2269803
anti-S6K	Cell Signaling Technology	CAT#9202S; RRID:AB_331676
anti-phospho-4E-BP1 Thr70	Cell Signaling Technology	CAT#9455; RRID:AB_330949
anti-4E-BP1	Cell Signaling Technology	CAT#9452S; RRID:AB_331692
anti-phospho-AKT S473	Cell Signaling Technology	CAT#4060S; RRID:AB_2315049
anti-AKT	Cell Signaling Technology	CAT#9272S; RRID:AB_329827
anti-phospho-PKC S657	Abcam	CAT#ab180848; RRID:AB_2783796
Anti-PKC	Cell Signaling Technology	CAT#2056T; RRID:AB_2284227
anti-Acetylated-Lysine	Cell Signaling Technology	CAT#9441L; RRID:AB_331805
anti-IRF3	Cell Signaling Technology	CAT#4302S; RRID:AB_1904036
anti-phosphoIRF3 Ser386	Cell Signaling Technology	CAT#37829S; RRID:AB_2799121
anti-CBP	Cell Signaling Technology	CAT#7389S; RRID:AB_2616020
anti-VSVG	Cell Signaling Technology	CAT#81454S
Anti-UL42	Abcam	CAT#ab19298; RRID:AB_444839
M2-conjugated magnetic beads	Sigma	CAT#A2220; RRID:AB_10063035
anti-Flag antibodies	Sigma	CAT#F1804; RRID:AB_262044
Anti-rabbit IgG (H+L) (DyLight™ 680 Conjugate)	Cell Signaling Technology	CAT#5366; RRID:AB_10693812
Anti-mouse IgG (H+L) (DyLight™ 800 Conjugate)	Cell Signaling Technology	CAT#5257; RRID:AB_10693543
Goat anti-Mouse IgG (H+L) Highly Cross-Adsorbed Secondary Antibody, Alexa Fluor 488	Introvigen	CAT#A11029; RRID:AB_2534088
Goat anti-Rabbit IgG (H+L) Cross-Adsorbed Secondary Antibody, Alexa Fluor 633	Introvigen	CAT#A21070; RRID:AB_2535731

**Bacterial and virus strains**		

BL21	Vazyme	CAT: C504-02
VSV-GFP	Dr. Hui Zheng Lab	N/A
HSV	Dr. Hui Zheng Lab	N/A

**Chemicals, peptides, and recombinant proteins**		

Rapamycin	Selleck	S1039
NAM	Selleck	S1899
TSA	Selleck	S1045
RC32	(Sun et al., 2019)	N/A
CI-994	Selleck	S2818
FK228	Selleck	S3020
RGF966	Selleck	S7229
LMK225	Selleck	S7569
Nexturastat A	Selleck	S7473
AGK2	Selleck	S7577
Nickel-agarose beads	Thermo Scientific.	CAT: #88221
Immobilized Glutathione	Thermo Scientific.	CAT: #15160
Lyso-Tracker Red	KeyGEN Biotech	CAT: KGMP006
ProLong™ Gold	Thermo Scientific	CAT: P10144
IRF3 peptide	This paper	N/A
Recombinant SIRT2 protein	This paper	N/A
Recombinant FKBP12 protein	This paper	N/A
Recombinant FRB protein	This paper	N/A
Recombinant HDAC1 protein	This paper	N/A
Recombinant IRF3 protein	This paper	N/A

**Experimental models: cell lines**		

A549	ATCC	RRID:CCL-185
HEK293T	ATCC	RRID:CRL-11268
MEF	Dr. Hui Zheng Lab	N/A
Hela	ATCC	RRID:CCL-2

**Recombinant DNA**		

Flag-FKBP12	This paper	N/A
HA-FKBP12-K45R	This paper	N/A
HA-FKBP12-K48R	This paper	N/A
HA-FKBP12-K48Q	This paper	N/A
HA-FKBP12-K53R	This paper	N/A
HA-FKBP12-K53Q	This paper	N/A
HA-FKBP12	This paper	N/A
Myc-FKBP12	This paper	N/A
Myc-Rheb	This paper	N/A
Flag-Rheb	This paper	N/A
Flag-Rheb-K8R	This paper	N/A
Flag-Rheb-K8Q	This paper	N/A
Flag-Rheb-K121R	This paper	N/A
Flag-Rheb-K121Q	This paper	N/A
Flag-mTOR	This paper	N/A
Myc-mTOR	This paper	N/A
mTOR-N-Luc	This paper	N/A
FKBP12-C-Luc	This paper	N/A
FKBP12-C-Luc-K45R	This paper	N/A
FKBP12-C-Luc-K45Q	This paper	
FKBP12-C-Luc-E108A	This paper	N/A
Rheb-N-Luc	This paper	N/A
Rheb-N-Luc-K8R	This paper	N/A
Rheb-N-Luc-K8Q	This paper	N/A
Rheb-N-Luc-K169R	This paper	N/A
Rheb-N-Luc-K169Q	This paper	N/A
HA-CBP	This paper	N/A
CBP	This paper	N/A
HA-P300	This paper	N/A
Myc-Tip60	This paper	N/A
Flag-Sirt2	This paper	N/A
Flag-Sirt2-H187Y	This paper	N/A
ISRE-Luc	This paper	N/A
Flag-IRF3	This paper	N/A
HA-Raptor	Dr. Jiahuai Han Lab	N/A

**Oligonucleotides**		

shRNA	Santa Cruz	CAT: sc-35378-SH
IFN-α Forward:5’-gtgaggaaatacttccacagactact-3′ Reverse:5′-tgaggaagagaaggctctcatga-3′	(Zhou et al., 2017)	N/A
IFN-βsequencing Forward:5′-cattacctgaaggccaagga-3′ Reverse:5′-cagcatctgctggttgaaga-3′	This paper	NA
ISG54 Forward:5′-cacctctggactggcaatagc-3′ Reverse: 5′-gtcaggattcagccgaatgg-3′	This paper	N/A
ISG56 Forward:5′-tacagcaaccatgag-3′ Reverse:5′-tcaggtgtttcacataggc-3′	This paper	N/A
UL46 Forward:5′-gcacccgttcaagcacaac -3′ Reverse: 5′-ccagtgagcagagtgacg -3′	(Ye et al., 2018)	N/A
ICP27 Forward:5′-cggctacagtatctgcgtca-3′ Reverse:5′-agccaccaggtcagagacat -3′	(Bergallo et al., 2008)	N/A
VSV Forward:5′-acggcgtacttccagatgg-3′ Reverse: 5′-ctcggttcaagatccaggt-3′	This paper	N/A
GAPDH Forward:5′-tcgacagtcagccgcatct-3′ Reverse:5′-ccgttgactccgaccttca-3′	This paper	N/A

**Software and algorithms**		

Prism	graphpad	https://www.graphpad.com/
Flowjo	NIH	https://imagej.nih.gov/ij/
Image J	NIH	https://imagej.net

### Resource availability

#### Lead contact

Further information and requests for resources and reagents should be directed to and will be fulfilled by the lead contact, Y. Eugene Chin (chinyue@suda.edu.cn).

#### Materials availability

All unique reagents generated in this study are available from the Lead Contact with a completed Material Transfer Agreement

### Experimental model and subject details

#### Cell line

The following cell line were used in the study

1. Human 293T Cell line (RRID: CRL-11268)

Origin: Human embryonic kidney tissue

Culture media and conditions:293T cells were obtained from ATCC and were cultured in Dulbecco's Modified Eagle's Medium with 10% fetal bovine serum (EallBio, 03.U16001DC), penicillin (100 U/ml), and streptomycin (100 mg/ml) in a humidified 5% CO_2_ incubator at 37°C. We did not authenticate this cell line in our laboratory.

2. A549 Cell line (RRID: CCL-185)

Origin: Human lung carcinoma cell line obtained from a 58-year-old male.

Culture media and conditions:A549 cells were obtained from ATCC and were cultured in Dulbecco's Modified Eagle's Medium with 10% fetal bovine serum (EallBio, 03.U16001DC), penicillin (100 U/ml), and streptomycin (100 mg/ml) in a humidified 5% CO_2_ incubator at 37°C. We did not authenticate this cell line in our laboratory.

4. Hela Cell line (RRID: CCL-2)

Origin: Human cervix adenocarcinoma cell line obtained from a 31-year-old female.

Culture media and conditions:Hela cells were obtained from ATCC and were cultured in Dulbecco's Modified Eagle's Medium with 10% fetal bovine serum (EallBio, 03.U16001DC), penicillin (100 U/ml), and streptomycin (100 mg/ml) in a humidified 5% CO_2_ incubator at 37°C. We did not authenticate this cell line in our laboratory.

### Method details

#### Cell culture

Amino acids starvation based method as being described, Briefly, cells were washed with PBS (Hyclone, SH30256.01B) and transferred into the prepared EBSS medium (Solabio, H2020) for 3 hrs.

HEK293T and HeLa cells were transfected with polyethyleneimine (Mr 40,000; Polysciences) or Lipofectamine 2000 (Invitrogen, 11668019) according to the manufacturers’ instructions. For production of lentiviral supernatants, HEK293T cells were cotransfected with the indicated lentiviral vectors together with helper plasmids using polyethyleneimine. Viral stocks were prepared by collecting the medium 48 hrs after transfection.

Stable cell lines with ectopic expression of FKBP12 and its mutants were established by lentivirus transduction. FKBP12-/- MEFs were infected with virus in the presence of polybrene (8 μg/ml). Twenty-four hrs after infection, media were changed to fresh medium containing 1 μg/ml puromycin. Five days later, the GFP positive cells were sorted for further experiments.

#### Co-immunoprecipitation (IP) and western blotting

Cells were treated or transfected as indicated in the figure legends and washed once with cold PBS. Cells were lysed in lysis buffer (20 mM Tris-HCl, pH 7.4, 150 mM NaCl, 0.5% NP-40, 10% glycerol, 1 mM DTT and complete protease inhibitor cocktail) for 15 min on ice and centrifuged at 20,000 g for 10 min. Protein was measured in the supernatants, and equal amounts of protein were processed for IP. For IP of endogenous FKBP12, 2 μg specific antibodies were routinely added into the soluble fraction, and rotated for 1 hr at 4°C. 20 μl of a 50% slurry of protein A/G-beads (Thermo Scientific, 20241) was incubated with soluble proteins overnight at 4°C. Immunoprecipitates were washed with PBS 3 times and subjected to western blotting with specific antibodies for analysis. For tag-protein IP, 5 μl of a 50% slurry of beads was used for each sample.

The prepared protein add 5×SDS and boil for 10minutes at 95°C,then samples were separated by using SDS-PAGE, transferred onto PVDF membranes and incubated with the corresponding antibodies. The bands were analyzed using Odyssey Infrared Imaging System.

#### Protein purification

GST-tagged SIRT2, HDAC1, mTOR (2011-2144 aa), FKBP12, and His-tagged FKBP12, Rheb were expressed in *E.coli* strain BL21 (C504-02) by induction with 0.1 mM isopropyl β-D-thiogalactoside for 12 h at 16°C. His-tagged proteins and GST-tagged proteins were purified using nickel-agarose beads and glutathione Sepharose beads, respectively, according to the manufacturers’ instructions. Flag-tagged FKBP12, Rheb, and IRF3 were transiently expressed in HEK293T cells for 36 hrs and purified using anti-Flag M2 magnetic beads (Sigma, M8823) according to the manufacturer’s instructions. Purified proteins were added to a final concentration of 10% glycerol and stored at -80°C.

#### Total RNA isolation and quantitative real-time PCR

Total RNAs were extracted using TRIzol (TIANGEN, DP-405-02) according to the manufacturer’s instructions. Total RNA (2 μg) was reverse transcribed using Prime Script RT reagent kit (TaKaRa, RR036A). Quantitative RT-PCR was carried out using SYBR green (Bimake, B21203) and ABI Step One Plus real-time PCR system (Applied Biosystems).

#### Luciferase reporter assay

The dual-element reporter assay system was performed based on a previously described method (Paulmurugan and Gambhir, 2007). Briefly, FKBP12 was fused to C-luc, and FRB or Rheb was fused to N-luc. Rapamycin was added to induce the approaching and reconstitution of split luciferase. HEK293T cells were transfected in triplicate with 100 ng of firefly luciferase reporter plasmids and 10 ng of sea pansy luciferase reporter plasmids (pTK-RL) using polyethylenimine in 24-well plates. Twenty four hours after transfection, cells were harvested and collected to measure firefly and sea pansy luciferase activities.

#### *In vitro* kinase assay

For peptide assay, mTOR complex purified from 293T cells (∼1000 nM) were incubated with 2 μg IRF3 peptide in the presence of 0.5 μM okadaic acid and ATP buffer (50 mM Tris -HCl, pH 7.5, 2 mM ATP, 5 mM MgCl2, 0.5 mM DTT) and incubated at 30°C for 60 minutes. The reaction mixture supernatant were then desalted and enriched using a μ-C18 Zip Tip (Millipore, ZTC18S096) and eluted directly onto an MALDI plate containing 2 mL CHCA (a-cyano-4-hydroxycinnamic acid) saturated solution in 50% acetonitrile (ACN) and 0.1% trifluoroacetic acid (TFA). MALDI-TOF spectra were acquired on a Voyager DE Promass spectrometer from Applied Biosystems.

For protein assay, mTOR complex purified from 293T cells (∼1000 nM) were incubated with 2 μg GST-IRF3 in the presence of 0.5 μM okadaic acid and ATP buffer (50 mM Tris -HCl, pH 7.5, 2 mM ATP, 5 mM MgCl2, 0.5 mM DTT) and incubated at 30°C for 60 minutes. The reaction mixtures were analyzed by western blot with indicated antibodies.

#### *In vitro* acetylation assay

For FKBP12 and Rheb *in vitro* acetylation assay, GST-FKBP12 (500 ng) or Rheb (500 ng) incubated with CBP(10 ng) in 50 mM Tris (pH7.9), 10% glycerol, 1 mM DTT, and 20 μM acetyl-CoA at 30°C for 60 min. The reaction mixtures were analyzed by western blot with indicated antibodies.

#### HPLC-MS/MS

To prepare samples for mass spectrometric analysis of acetylation site(s) of FKBP12 and Rheb, immunoprecipitated proteins from HEK293T cells were separated by SDS-PAGE and visualized using Coomassie blue staining. Following reduction and alkylation, in-gel digestion was performed with trypsin (Thermo Scientific, 90058) at 37°C for 16 hrs. The peptides were desalted and enriched using a μ-C18Zip Tip (Millipore) and eluted into 1% trifluoroacetic acid in 50% acetonitrile aqueous solution. The extracts were then combined and dried in a Speedvac. For LC-MS/MS analysis, the tryptic digested peptides were separated by EASY nLC 1200, and the eluted peptides were ionized and directly introduced into Orbitrap Fusion Lumos (Thermo Fisher Scientific). The data were analyzed using Proteome Discoverer 2.2.

#### ITC measurements

ITC (Isothermal Titration Calorimetry) experiments were performed at 20°C using MicroCalPEAQ-ITC (Malvern Panalytical Ltd, United Kingdom) for label-free solution measurements of the binding affinity (Kd) and stoichiometry (n) between rapamycin and the recombinant proteins. Rapamycin powder (Selleck, S1039) was dissolved in phosphate buffered saline (137 mM NaCl, 2.7 mM KCl, 10 mM Na_2_HPO_4_, 2 mM KH_2_PO_4_) containing 4.6% DMSO to final concentration of 100 μM. Protein samples were conditioned in the same buffer at final concentrations ranging from 3 to 9 μM (calculated from their UV_280_ nm using the sequence-estimated extinction coefficient). The integration of heats was adjusted manually. The ITC isotherm(s) were fitted using a one-site-binding model.

#### Microscale thermophoresis assay

A Monolith NT Automated from NanoTemper Technologies was used for MST assays. FRB, and HDAC1 proteins were fluorescently labeled with the RED-tris-NTA (MO-L008) according to the manufacturer’s instructions. SIRT2 was carried out by the LabelFree method as previously described (Soares da Costa et al., 2016). All affinity measurements were performed in PBS buffer mixed with 0.05% Pluronic F-127 (Invitrogen, P6866). Rapamycin, arrayed at different concentrations, was incubated with proteins for 30 min before application to Monolith NT standard treated capillaries. Thermophoresis was then determined at 25°C with 15-20% excitation power and middle MST power.

#### Immunofluorescence

The prepared cells were washed with PBS two times, then fixed with 4% paraformaldehyde in phosphate-buffered saline (PBS) for 10 min, then incubated in blocking buffer (3% BSA in PBS) for 1h at RT. The cells were then incubated with primary antibodies in blocking buffer overnight, washed three times, and incubated with secondary antibodies for 1h at RT. The cells were subsequently stained with 1 μg/ml Hoechst in PBS for 10 min, washed three times, and mounted with ProlongGold (Thermo Fisher Scientific). All sliders were examined using a Nikon Eclipse Ti confocal microscope.

### Quantification and statistical analysis

Data are presented as mean ± standard deviation (SD), and statistical significance are reported in the figure legends. Statistics was analysis using Student’s *t* test, or analysis of variance (ANOVA) analysis (one-way ANOVA for comparisons between groups). p-values were shown on the figures as asterisks: ∗, p < 0.05; ∗∗, p < 0.01; ∗∗∗, p < 0.001; ∗∗∗∗, p < 0.0001. Independently performed biological replicates (n values) are indicated as circles in the bar graphs.

## Data Availability

All data reported in this paper will be shared by the lead contact upon request. This paper does not report original code. Any additional information required to reanalyze the data reported in this paper is available from the lead contact upon request.
