# Supplementary material for: Rapamycin recruits SIRT2 for FKBP12 deacetylation during mTOR activity modulation in innate immunity
Source: iScience. 2021 Sep 27;24(11):103177. doi: 10.1016/j.isci.2021.103177 (PMC8529501; doi:10.1016/j.isci.2021.103177)
Supplement: Document S1. Figures S1–S6 [file mmc1.pdf]

iScience, Volume *24*

Supplemental information

Rapamycin recruits SIRT2 for FKBP12 deacetylation during mTOR activity modulation in innate immunity

Lin Hu, Fuxian Chen, Chao Wu, Jun Wang, Si-si Chen, Xiang-rong Li, Jing Wang, Linpeng Wu, Jian-ping Ding, Jian-chuan Wang, Chao Huang, Hui Zheng, Yu Rao, Yu Sun, Zhijie Chang, Wei Deng, Cheng Luo, and Y. Eugene Chin

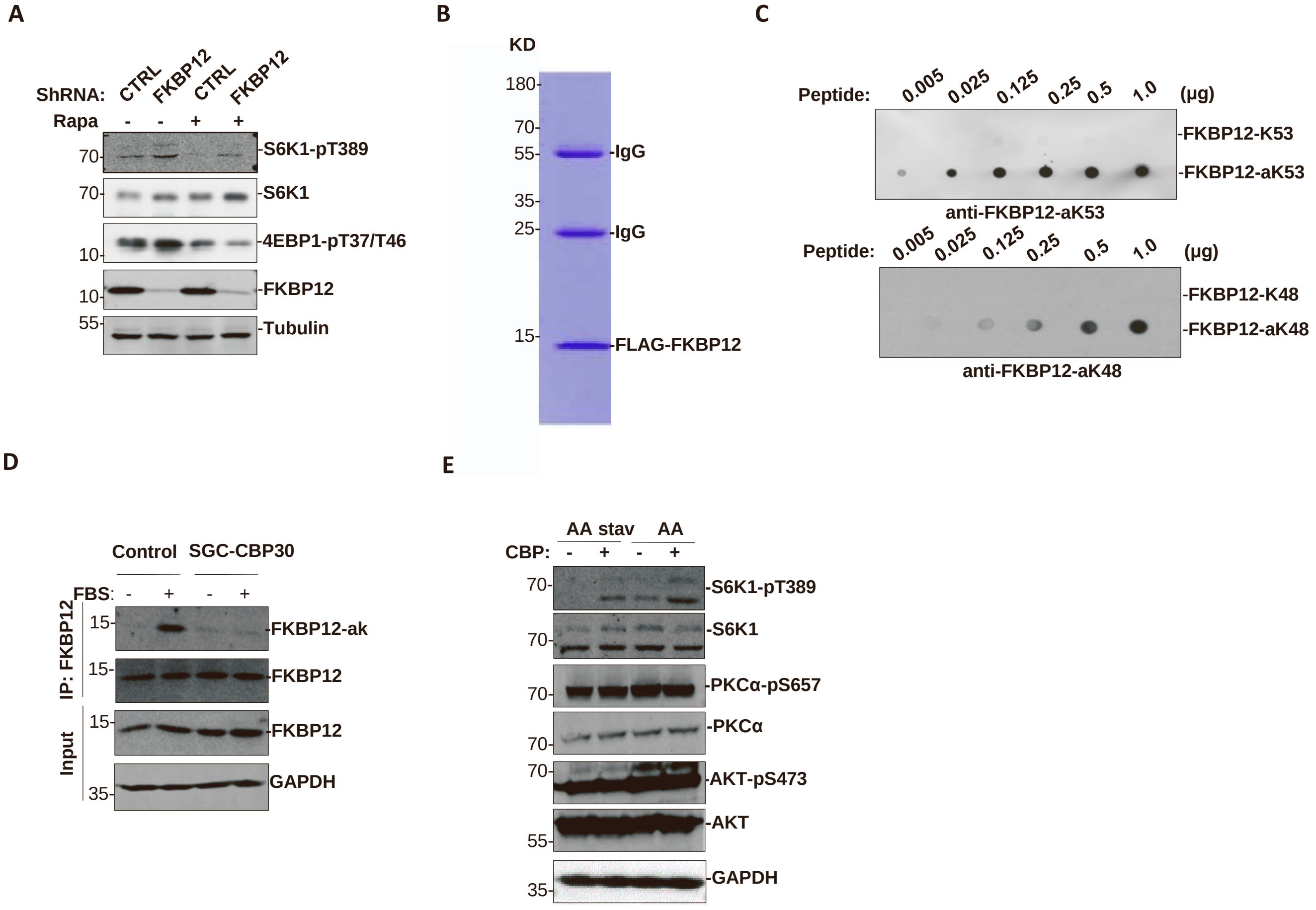

Supplementary Figure. 1 Depletion of FKBP12 induces S6K1 phosphorylation(Related to Figure 1).

A In A549 cells, control (CTRL) or FKBP12 shRNA was introduced followed by rapamycin (100 nM) treatment or without treatment for 30 min, WCLs were prepared and submitted to Western blot with anti-S6K1-pT389 and 4EBP1 phosphorylation respectively.

B Commassie blue staining showing the purity of FLAG-FKBP12 obtained from HEK293T cells transfected with FLAG-FKBP12 and CBP.

C Polyclonal antibodies prepared from rabbits were analyzed with dot blotting assay. Immobilon NC tansfer membrane was spotted with different doses as indicated for non-acetyl and acetyl-peptides of FKBP12 K53 or K48.

D Hela cells were treated by SGC-CBP30 at a concentration of 2μM for 6 hrs prior to FBS stimulation, WCLs were prepared and submitted to western blot with indicated antibodies.

E In HEK293T cells with or without CBP overexpression, AA starvation for 24 hrs was followed by amino acids treatment for 30 min, mTOR activity was analyzed by blotting with anti-S6K1-pT389 or anti-AKT-pS473.

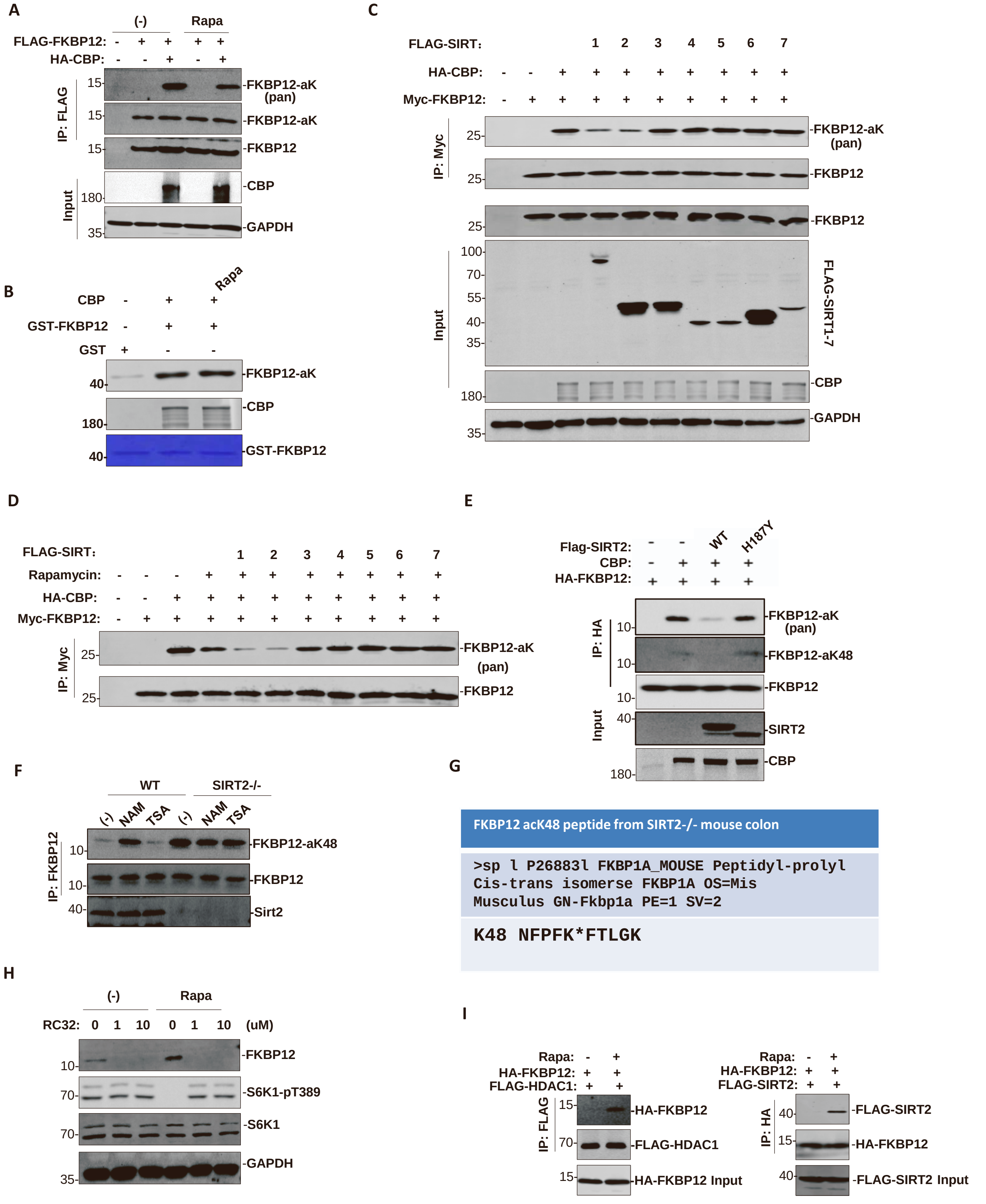
A
(-)
Rapa
FLAG-FKBP12: - + + + +
HA-CBP: - - + - +
IP: FLAG
Input
FKBP12-aK (pan)
FKBP12-aK
FKBP12
CBP
GAPDH
B
Rapa
CBP - + +
GST-FKBP12 - + +
GST + - -
FKBP12-aK
CBP
GST-FKBP12
C
FLAG-SIRT: 1 2 3 4 5 6 7
HA-CBP: - - + + + + + + + +
Myc-FKBP12: - + + + + + + + + +
IP: Myc
Input
FKBP12-aK (pan)
FKBP12
FLAG-SIRT1-7
CBP
GAPDH
D
FLAG-SIRT: 1 2 3 4 5 6 7
Rapamycin: - - - + + + + + + + +
HA-CBP: - - + + + + + + + + +
Myc-FKBP12: - + + + + + + + + + +
IP: Myc
FKBP12-aK (pan)
FKBP12
E
Flag-SIRT2: - - WT H187Y
CBP: - + + +
HA-FKBP12: + + + +
IP: HA
Input
FKBP12-aK (pan)
FKBP12-aK48
FKBP12
SIRT2
CBP
F
WT
SIRT2-/-
(-) NAM TSA (-) NAM TSA
IP: FKBP12
FKBP12-aK48
FKBP12
Sirt2
G
FKBP12 acK48 peptide from SIRT2-/- mouse colon
>sp l P26883l FKBP1A_MOUSE Peptidyl-prolyl
Cis-trans isomerse FKBP1A OS=Mis
Musculus GN-Fkbp1a PE=1 SV=2
K48 NFPFK*FTLGK
H
(-)
Rapa
RC32: 0 1 10 0 1 10 (uM)
FKBP12
S6K1-pT389
S6K1
GAPDH
I
Rapa: - +
HA-FKBP12: + +
FLAG-HDAC1: + +
IP: FLAG
HA-FKBP12
FLAG-HDAC1
HA-FKBP12 Input
Rapa: - +
HA-FKBP12: + +
FLAG-SIRT2: + +
IP: HA
FLAG-SIRT2
HA-FKBP12
FLAG-SIRT2 Input

Supplementary Figure. 2 SIRT and HDAC members deacetylate FKBP12 with distinct mechanisms(Related to Figure 2).

A In HEK293T cells, FKBP12 was transfected alone or with CBP followed by no treatment or rapamycin treatment. Anti-Flag IPs were analyzed in Western blot with pan anti-acetyl-K for FKBP12 acetylation.

B Acetylation of FKBP12 by CBP treated with or without rapamycin in vitro acetylation assay.

C-D All SIRT family members (SIRT 1-7) tagged in Flag were cotransfected with CBP in HEK293T cells followed by rapamycin treatment or not. Anti-FKBP12 IPs were analyzed in western blot for FKBP12 acetylation with pan anti-acetyl-K.

E SIRT2 wild type (WT) and SIRT2 catalytic inactive form (H187Y) on FKBP12 deacetylation in HEK293T transfectants.

F In WT and SIRT2-/- MEFs, FKBP12 acetylation induction by TSA or NAM or no treatment (-) was analyzed in western blot by using pan anti-acetyl-K.

G Using differential TMT analysis of fibroblasts obtained from WT and SIRT2-/- mouse liver, FKBP12 K48 acetylation peptide was recovered from SIRT2-/- fibroblasts.

H HeLa cells were treated with indicated doses of RC32 for 12 hrs followed by no treatment or rapamycin treatment for 1hrs. WCLs were analyzed with indicated antibodies.

I HEK293T cells expressing ectopic Flag-HDAC1 and HA-FKBP12 were treated with or without rapamycin. Anti-Flag IPs from WCLs were analyzed with anti-HA for FKBP12 and HDAC1 interaction in western blot (left panel). HEK293T cells expressing ectopic Flag-SIRT2 and HA-FKBP12 were treated with or without rapamycin. Anti-Flag IPs from WCLs were analyzed with anti-HA for FKBP12 and SIRT2 interaction in western blot.

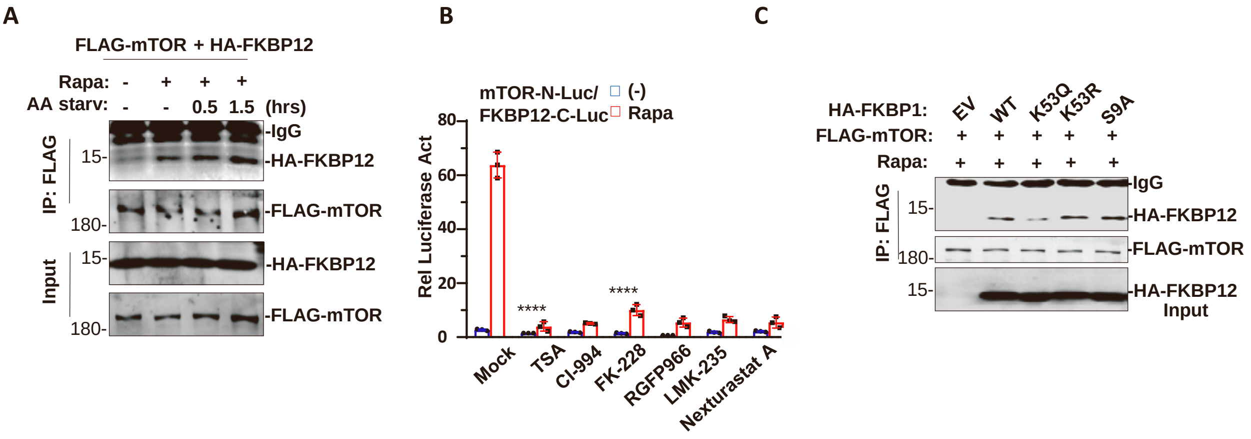

Supplementary Figure. 3 FKBP12 and mTOR association can be disrupted by HDAC inhibitors(Related to Figure 3).

A Flag-mTOR and HA-FKBP12 were cotransfected in HEK293T cells. Amino acids starvation was followed by rapamycin treatment. Anti-Flag IPs were obtained from WCLs followed by western blot analysis with anti-FLAG and anti-HA for mTOR and FKBP12 interaction.

B mTOR-N-luc and FKBP12-C-luc of two reporter system were transfected in HEK293T cells. Cells were treated with indicated HDAC inhibitors for 6 hrs and followed by rapamycin treatment for additional 6 hrs. Luciferase activity was then analyzed with WCLs. Data are represented as mean $\pm$ SD; ****, $p<0.0001$, one-way ANOVA.

C In HEK293T cells, FLAG-mTOR was cotransfected with HA-FKBP12 variants (K53Q, K53R an S9A) followed by rapamycin treatment. Anti-Flag (mTOR) IPs from WCLs were analyzed in western blot with anti-HA (FKBP12) for mTOR and FKBP12 interaction.

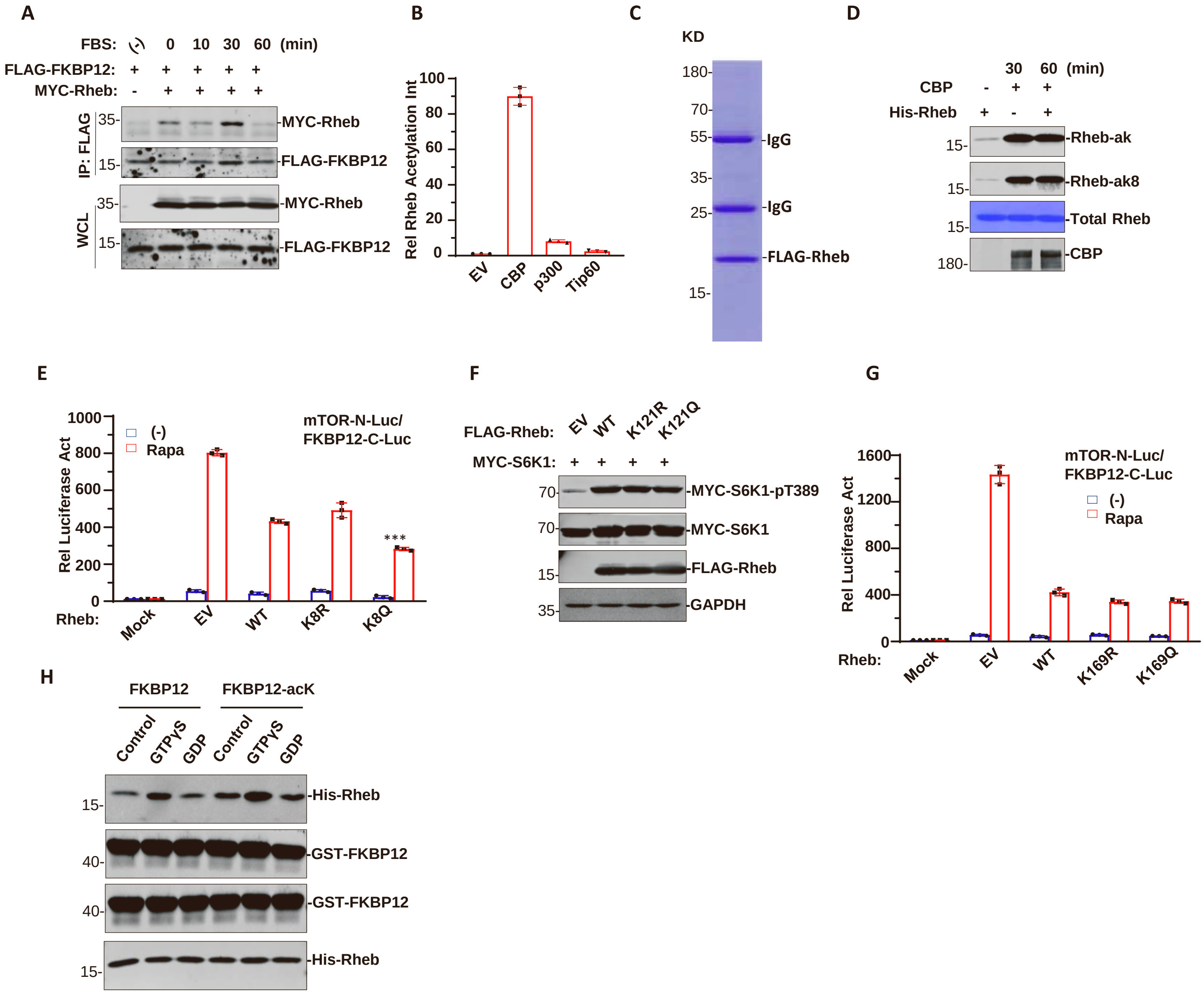

Supplementary Figure. 4 Acetyl-FKBP12 and acetyl-Rheb association for mTOR activation(Related to Figure 4).

A Flag-FKBP12 alone or together with Myc-Rheb were transfected in HEK293T cells followed by no or FBS treatment for indicated times. Anti-Flag (FKBP12) IP was analyzed with anti-Myc (Rheb) for FKBP12 and Rheb interaction.

B Flag-Rheb was transfected with CBP, p300 or Tip60 in HEK293T cells. Anti-Flag IPs were detected with pan acetyl-K antibody (mono). The acetylation intensity was plotted. Data are represented as mean± SD.

C Commassie blue staining to show the purity of FLAG-Rheb obtained from HEK293T cells transfectants of FLAG-Rheb and CBP.

D Bacterially expressed His-Rheb and CBP were subjected to in vitro acetylation assay.

E Two reporter system (mTOR-N-Luc and FKBP12-C-Luc) was transfected along with Rheb-WT, K8R or K8Q in HEK293T cells. After rapamycin treatment for 48 hrs, WCLs were prepared for luciferase activity analysis. Data are represented as mean ± SD; ***, p<0.001, one-way ANOVA.

F Flag-Rheb WT, K121R, and K121Q were transfected along with Myc-S6K1 in HEK293T cells. S6K1 phosphorylation was analyzed in western blot with specific anti-S6K1 pT389.

G Two reporter system (mTOR-N-luc and FKBP12-C-Luc) was cotransfected along with Rheb WT, K169R, or K169Q. After rapamycin treatment for 48 hrs, WCLs were prepared for luciferase activity analysis. Data are represented as mean ± SD.

H Untreated Rheb, GTPγS-loaded Rheb, and GDP-loaded Rheb were all incubated with GST-FKBP12 followed by precipitation with glutathione beads. The precipitates were analyzed in western blot with indicated antibodies.

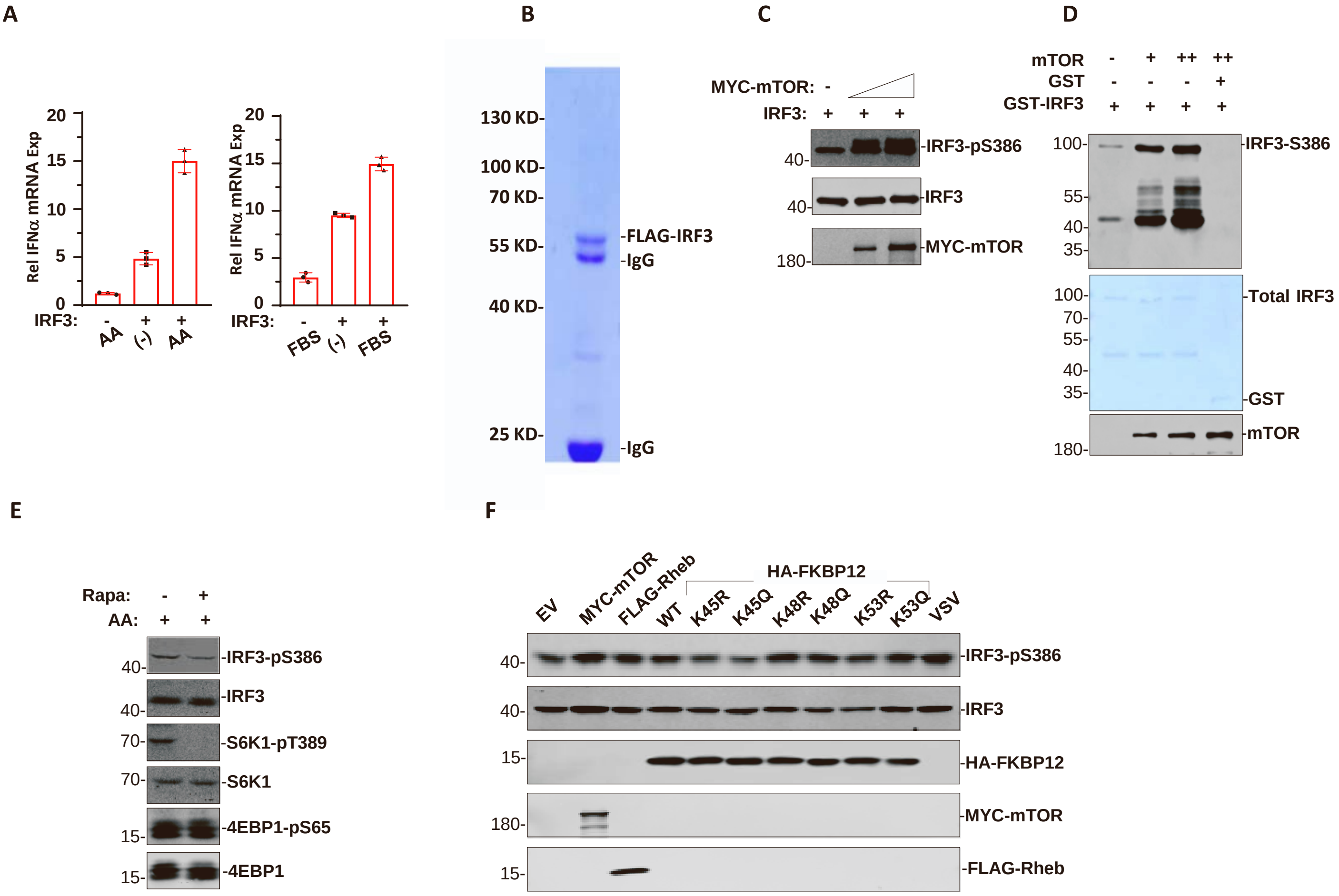

Supplementary Figure. 5 IRF3 phosphorylation is regulated by mTOR(Related to Figure 5).

A HEK293T cells were transfected with or without IRF3 as indicated followed by AA treatment or FBS treatment. RT-PCR was performed to analyze IFNβ mRNA expression level. Data are represented as mean ± SD.

B Flag-IRF3 protein was purified from HEK293T cells transfected with Flag-IRF3 and mTOR by using Flag-beads.

C IRF3 S386 is phosphorylated by Myc-mTOR with increasing amount in HEK293T cells.

D mTORC1 phosphorylates IRF3 at S386 in an vitro assay.

E FBS and amino acids starved fibroblasts were treated with amino acids followed by with or without rapamycin (100 nM) treatment for 15 min. WCLs were prepared for IRF3, S6K1, or 4EBP1 phosphorylation analysis in western blot with anti-IRF3-pS386, S6K1-pT389 and anti-4EBP1-pS65 respectively.

F In 2fTGH human fibroblasts, mTOR，Rheb as well as indicated FKBP12 variants were transfected for IRF3 S386 phosphorylation induction. IRF3-S386 phosphorylation was detected in western blot with anti-IRF3-pS386.

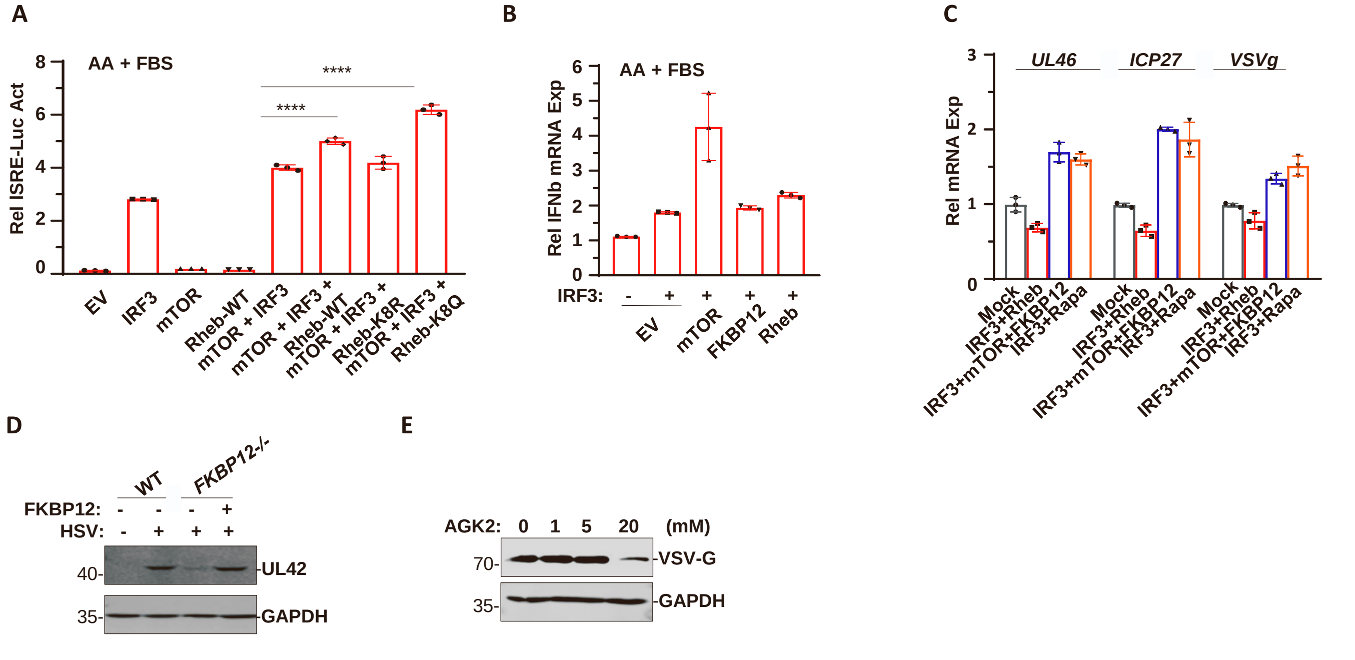

Supplementary Figure. 6 mTOR-IRF3 pathway triggers antiviral response(Related to Figure 6).

A In HEK293T cells, IRF3, mTOR and Rheb or Rheb variants alone or combined as indicated were tested for IRF3 binding promoter ISRE-luciferase activity induction. Data are represented as mean $\pm$ SD; ****, p<0.0001, one-way ANOVA.
B In HEK293T cells, IRF3 was alone or cotransfected with mTOR, FKBP12, or Rheb. IFNβ mRNA expression was analyzed with RT-PCR. Data are represented as mean $\pm$ SD.
C In human fibroblasts (2fTGH) cells, empty vector, IRF3 was cotransfected with Rheb or IRF3 was cotransfected with Rheb and FKBP12 as indicated. HSV virus products UL46 and ICP27 and VSV virus product VSVg were detected by performing RT-PCR. Data are represented as mean $\pm$ SD.
D FKBP12-/- mouse fibroblasts were transiently transfected with or without FKBP12 and compared with wild type fibroblasts for HSV infection. HSV virus protein UL-42 was analyzed in western blot.
E Fibroblasts cells were infected with VSV-GFP followed by AGK2 treatment as indicated concentration and the cells were analyzed by western blot with anti-VSV-G.
